# Pediatric andrology: the andrological patient from infancy to adulthood. Prevention interventions: how and when

**DOI:** 10.1186/1824-7288-41-S2-A10

**Published:** 2015-09-30

**Authors:** Salvatore Bonforte

**Affiliations:** 1 Pediatra di famiglia ASP/3 Catania, Italy

## 

Sonography, a non-invasive, quick method without biological impact on the gonad, has greatly improved the diagnostic possibilities; it is the gold standard for the male genital system (gonads and genito-urinary tract) and in fact has oriented the prevention interventions [1].

It recognizes almost 100% of scrotal lesions and their solid, liquid or complex nature. It has allowed to overcome the limits of traditional diagnostic methods for gonads like palpation, transillumination, and the Prader orchidometer for volumetric assessment. In fact, compared to this, sonography allows a more accurate volume measurement, expecially in small testicles; a correlation between measurements made with the orchidometer and those made with ultrasounds was found only for testicular volumes over 4 cc. [2].

Measurement of testicular volume is a fundamental element in pediatric andrology for evaluating puberty onset and progression. Furthermore, the sonographic exam is one of the best tools for assessing testicular pathologies like torsion, undescended testis, varicocele and in general in pathologies involving male genitalia; these cases can show significant variations of testicular volume. Testicular growth restriction may have relevant clinical implications for future testicular function [3].

The limits of traditional tools, like the orchidometer or the ruler for the measurement of pre-puberal testicle, are easy to identify:

- The smallest pearl in Prader orchidometer is 1 ml, while sonography allows to make measurements in the first years of life (0,44 ml ± 0,03);

- Orchidometer and ruler are known for overestimating testicular volume because they measure not only the didymus but also the epididymis (which in the first childhood is relatively big, compared to total testicular volume) and the scrotal tissues.

Testicular volume is related to many reproductive endocrine parameters; therefore, a measurement of testicular volumes with a reliable method is appropriate, because any scrotal anomaly can influence testicular growth, and should be detected and treated as soon as possible.

Moreover, andrological sonography is a first choice exam for male genitalia pediatric pathologies like acute scrotum, where diagnosis and clinical evaluations are particularly difficult. For example, during a suspected testicular torsion, a measurement made with a high resolution ultrasound machine by an expert examinator allows to reach a sensibility of 98% and a specificity of 99%, like scintigraphy and MRI with contrast medium.

The sonographic study of the testicular region has absolute indications and relative indications.

Absolute indications: difficult/inadequate objective clinical examination (sore and/or swelling testis, suggesting an acute scrotum; testicular torsion, trauma, undescended testis) or suspected testicular mass [4].
